# The effect of intraoperative radiotherapy in musculoskeletal malignancy: A population study from US SEER database

**DOI:** 10.7150/jca.100678

**Published:** 2025-01-01

**Authors:** Yao Xu, Zhe Feng, YongHeng Liu, JinYan Feng, Yan Zhang, Chao Zhang, XiuXin Han, LiMing Xu, GuoWen Wang

**Affiliations:** 1 Department of Bone and Soft Tissue Tumors, Tianjin Medical University Cancer Institute &Hospital, National Clinical Research Center for Cancer, Tianjin's Clinical Research Center for Cancer, Key Laboratory of Cancer Prevention and Therapy, Tianjin, Tianjin 300060, China.; 2 Department of Radiation Oncology, Tianjin Medical University Cancer Institute &Hospital National Clinical Research Center for Cancer, Tianjin's Clinical Research Center for Cancer, Key Laboratory of Cancer Prevention and Therapy, Tianjin, Tianjin 300060, China.

**Keywords:** Bone Sarcoma, Soft Tissue Sarcoma, Intraoperative Radiotherapy, SEER Program, Survival Outcome

## Abstract

**Objective:** The aim of our study was to explore the effect of IORT on survival outcome of patients with musculoskeletal malignancy. The prognostic factors of patients with IORT treatment were also identified in this study.

**Methods:** The retrospective analysis was conducted based on the Surveillance, Epidemiology, and End Results (SEER) database spanning from 2000 to 2020. The musculoskeletal malignancy patients who received both surgery and radiation therapy (RT) treatment were included into the study. Survival differences between groups were explored by Kaplan-Meier method and log-rank test. Potential prognostic factors of patients with IORT treatment were identified by Cox proportional hazards regression analysis.

**Results:** A total of 24,297 patients were selected finally, including 23,877 cases with neoadjuvant/adjuvant RT alone, 190 cases with IORT alone, and other 230 cases received both neoadjuvant/adjuvant RT and IORT. The median survival time of these patients was 141.0 (95%CI: 101.1-180.9) months. Patients who received both IORT and neoadjuvant/adjuvant RT treatment presented the best survival outcome when compared with those underwent either IORT or neoadjuvant/adjuvant RT only. Further subgroup analyses verified the survival benefit of the combination of IORT and neoadjuvant/adjuvant RT in female patients with tumor located on limb and in patients who received the performance of chemotherapy. A series of variables, including age at diagnosis, gender, primary tumor site, tumor Grade, SEER stage, T stage, N stage, IORT only or the combination of IORT and neoadjuvant/adjuvant RT, the performance of chemotherapy, were identified as independent prognostic factors of patients with IORT treatment.

**Conclusions:** The current study is distinguished by its large-scale analysis of the SEER database, encompassing a comprehensive cohort of musculoskeletal malignancy patients treated with IORT, as well as the rigorous subgroup analysis. We concluded that IORT during surgery procedure, accompanied with neoadjuvant/adjuvant RT, might confer a survival benefit for selected patients diagnosed with musculoskeletal malignancy.

## Introduction

Malignant musculoskeletal neoplasms, originating from bone or soft tissues, mainly included bone sarcomas and soft tissue sarcomas (STS), which accounting for nearly 1.0% of all adult tumors and about 15.0% in childhood 1. The incidence of bone sarcomas was reported to be 0.8-0.9 per 100,000 persons while the incidence of STS was up to 1.28-1.72 per 100,000 persons 2, 3. The prognosis of musculoskeletal malignancy was discrepant across numerous pathological subtypes due to obvious heterogeneity of histological manifestations. Nearly 9.0%-10.0% of patients would present local recurrence after tumor resection and about 7.5% of patients died of distant metastasis 3. It was reported that the relative survival of bone sarcomas at 3-year, 5-year and 10-year were 73.3%, 67.4% and 61.9%, respectively 4. The overall survival (OS) rate of low-grade sarcoma and high-grade sarcoma at 5 years were 87.0% and 62.0%, respectively, in STS 5.

The surgical resection was the mainstay of treatment in musculoskeletal malignancy and the 5-year disease-free survival rate after surgery was 50.0% as previously reported 6. Nevertheless, a variety of factors could determine the therapeutic efficacy of surgery, including tumor size, anatomic tumor location, and infiltration of surrounding vessels and organs 7. Moreover, the general condition of patients, as well as mode of surgery and surgical skills were of crucial importance to prognosis and outcomes 8. In addition, margin status was one of the most crucial risk factors affecting local tumor recurrence. Patients with negative margin (R0 resection) can usually achieve long-term local control while in cases with R1 or R2 resection, the rate of local recurrence was significantly increased 9, 10. The therapeutic principle of musculoskeletal malignancy has changed from amputation and similar radical surgical resection to a more comprehensive multi-modality way, and surgical wide resection plus RT has emerged as the standard approach for high-grade STS and specific types of bone sarcomas. According to the NCCN guidelines, neoadjuvant RT can be performed in patients with large high-grade STS to downstage the tumors while postoperative RT should be given in STS when the oncologically appropriate margins cannot be achieved 1.

Ionizing radiation could cause cell death by cleaving DNA 11. However, the killing effect of radiation on cells was not selective. While killing tumor cells, normal tissues in the radiation target area could also be damaged. The adequate radiation dose is not always achievable with traditional external beam radiotherapy (EBRT) methods, primarily due to the low radiation tolerance of adjacent normal organs and tissues in target area 12, 13. With the development of radiotherapy technology, three-dimensional radiotherapy methods, such as stereotactic body radiotherapy (SBRT), has become the main auxiliary approach to surgical treatment 14. Nevertheless, these radiotherapy methods could only be carried out in batches outside the perioperative period when the patient's performance status is favorable. While prolonging the treatment time, it will make both normal cells and tumor cells recover to a certain extent 15. Since the beginning of modern intraoperative radiotherapy (IORT) in the 1980s, growing evidence has demonstrated that IORT offers unique therapeutic advantages over traditional radiotherapy methods in musculoskeletal malignancy 16. A previous study indicated that the success rate of IORT in STS was up to 90.0% 17. In Germany, a total of 153 patients with limb STS were retrospectively analyzed and all the patients were received the treatment of intraoperative electron boost radiotherapy (IOERT) followed by EBRT 18. After a median follow-up of 33 months, the 5-year OS rate and 5-year local control rate were 77.0% and 78.0%, respectively 18. Nearly 23.0% of patients presented acute toxicity levels 2-4, and 17.0% had advanced toxicity level 2-4^18^. It concluded that the surgical resection combined with IORT not only improved the tumor control rate, but also resulted in acceptable radiation toxicity 18.

The Surveillance, Epidemiology, and End Results (SEER) database is one of the most representative large-scale oncology registration databases in North America, which included patients' demographic and clinicopathological characteristics as well as survival outcome information. Notably, the radiation modalities of each patient were recorded in detail. Thus, our study aimed to explore the characteristics of musculoskeletal malignancy patients who received IORT treatment based on data from the SEER database. Furthermore, we investigated the effect of IORT on survival outcome and identified the prognostic factors of musculoskeletal malignancy patients who were treated with IORT.

## Materials and Methods

### Data sources of SEER database

In the current study, we retrieved the clinical data of patients with musculoskeletal malignancy from the SEER database. The specific name of the database we used in the current study was illustrated as following: Surveillance, Epidemiology, and End Results (SEER) Program (www.seer.cancer.gov) SEER*Stat Database: Incidence - SEER Research Data, 17 Registries, Nov 2022 Sub (2000-2020) - Linked To County Attributes - Time Dependent (1990-2021) Income/Rurality, 1969-2021 Counties, National Cancer Institute, DCCPS, Surveillance Research Program, released April 2023, based on the November 2022 submission.

### Cohort selection and exclusion criteria of SEER analysis

We identified musculoskeletal malignancy patients diagnosed between 2000 to 2020 based on the variable 'AYA site recode 2020 Revision' in SEER*Stat software. The option was restricted to '4.Sarcomas', thus, including sarcomas originating both from soft tissue (soft tissue sarcoma category) and bone (bone sarcoma category) into the study. The soft tissue sarcoma category contained the following tumor types as SEER*Stat software recording: Ewing family of tumors originated from soft tissue, Fibromatous neoplasms, Liposarcoma, Synovial sarcoma, Leiomyosarcoma, Rhabdomyosarcoma, Gastrointestinal stromal tumor, Spindle cell sarcoma, Epithelioid sarcoma, Desmoplastic small round cell tumor, Giant cell sarcoma, Other soft tissue sarcomas. While the bone sarcoma category consisted of the following pathological types: Osteosarcoma, Chondrosarcoma, Ewing family of tumors originated from bone, Chordoma, Other bone tumors.

Initially, a total of 116,753 cases were selected. Patients diagnosed at autopsy, or indicated in death certification, and patients with unknown age information were routinely excluded. In order to exclude patients who did not receive either radiation therapy or surgical intervention, the variable 'RX Summ--Surg/Rad Seq' and the variable 'RX Summ--Surg Prim Site' were used. Finally, a total of 24,297 patients were included into the present study. Among them, 23,877 cases received pre/postoperative RT without IORT, thus deemed as neoadjuvant/adjuvant RT group. While the other 420 cases received IORT alone (IORT- group) or the combination of IORT and neoadjuvant/ adjuvant RT (IORT+ group). The flowchart of cohort selection was shown in supplementary Figure 1.

### Outcome Measures and statistics analysis

All the quantitative data were expressed as mean ± standard deviation (SD), while the categorical data were presented as the number and the percentage (N, %). Pearson chi-square (χ2) test or Fisher's exact test was used to evaluate the difference between categorical variables. The main outcome of the study was overall survival (OS), which was defined as the interval time between initial diagnosis and all cause of death or the last follow-up. The Kaplan-Meier method was performed and the log-rank test was employed to analyze survival difference between groups. To identify the prognostic factors of patients who received IORT, the Cox proportional hazards regression analysis was performed. Those variables with P<0.05 in univariate analysis were analyzed in a further multivariate analysis to determine the independent prognostic factors. The SPSS 26.0 (IBM Corporation, Armonk, NY) was used for statistical analyzing and all the survival curves were performed by MedCalc® Statistical Software version 20.100 (MedCalc Software Ltd, Ostend, Belgium; https://www.medcalc.org; 2022). All tests were two sided and values of P<0.05 were considered statistically significant.

### Ethics Statement

The present study is in accordance with the 1964 Declaration of Helsinki and subsequent amendments or comparable ethical standards. The informed consent of patients was not required since the SEER dataset is an open access database available all over the world and cancer a is reportable disease in the USA.

## Results

### Characteristics of the patients with musculoskeletal malignancy

**Figure 2:**
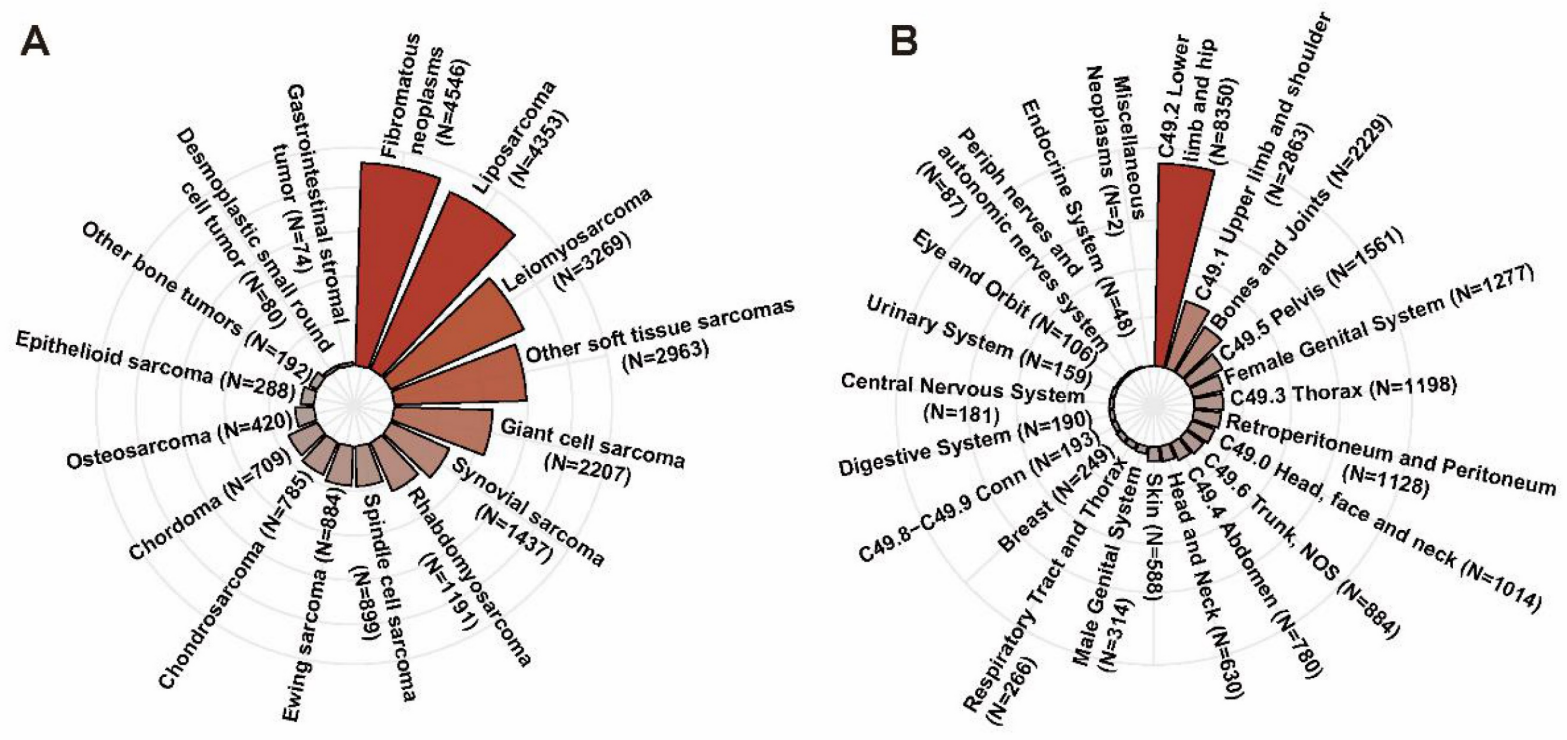
The distribution of cases in different pathologic types (A) and the distribution of cases in different primary tumor sites (B).

According to the inclusion and exclusion criteria, a total of 24,297 patients diagnosed with musculoskeletal malignancy in the SEER dataset were included. The mean age of all patients was 54.5±20.7 years with a slightly male predominance (N=13,030, 53.6%). As shown in Figure 2A, the top five pathologic types were fibromatous neoplasms (N=4,546, 18.7%), liposarcoma (N=4,353, 17.9%), leiomyosarcoma (N=3,269, 13.5%), giant cell sarcoma (N=2,207, 9.1%) and synovial sarcoma (N=1,437, 5.9%). As for primary tumor location, lower limb and hip was the most common tumor site accounting for 34.4% (N=8,350), followed by upper limb and shoulder (N=2,683, 11.8%), bones and joints (N=2,229, 9.2%). The distribution of cases in different primary tumor sites was described in Figure 2B.

Among all patients, a total of 23,877 patients were collected into the RT group, in which patients received both surgery without IORT and neoadjuvant/adjuvant RT. The other 420 patients received IORT during the surgical procedure. Detailed information about the baseline demographic and clinicopathological characteristics in the RT group and the IORT group were presented in Table 1. The distribution of gender, marital status, SEER stage and distant metastases were not statistically different between the RT group and the IORT group. It seemed that patients in the IORT group possessed a higher level of income than the RT group. Besides, the primary tumor site of limb was more common in IORT group and these patients were more tended to undergo chemotherapy at the same time.

**Figure 3:**
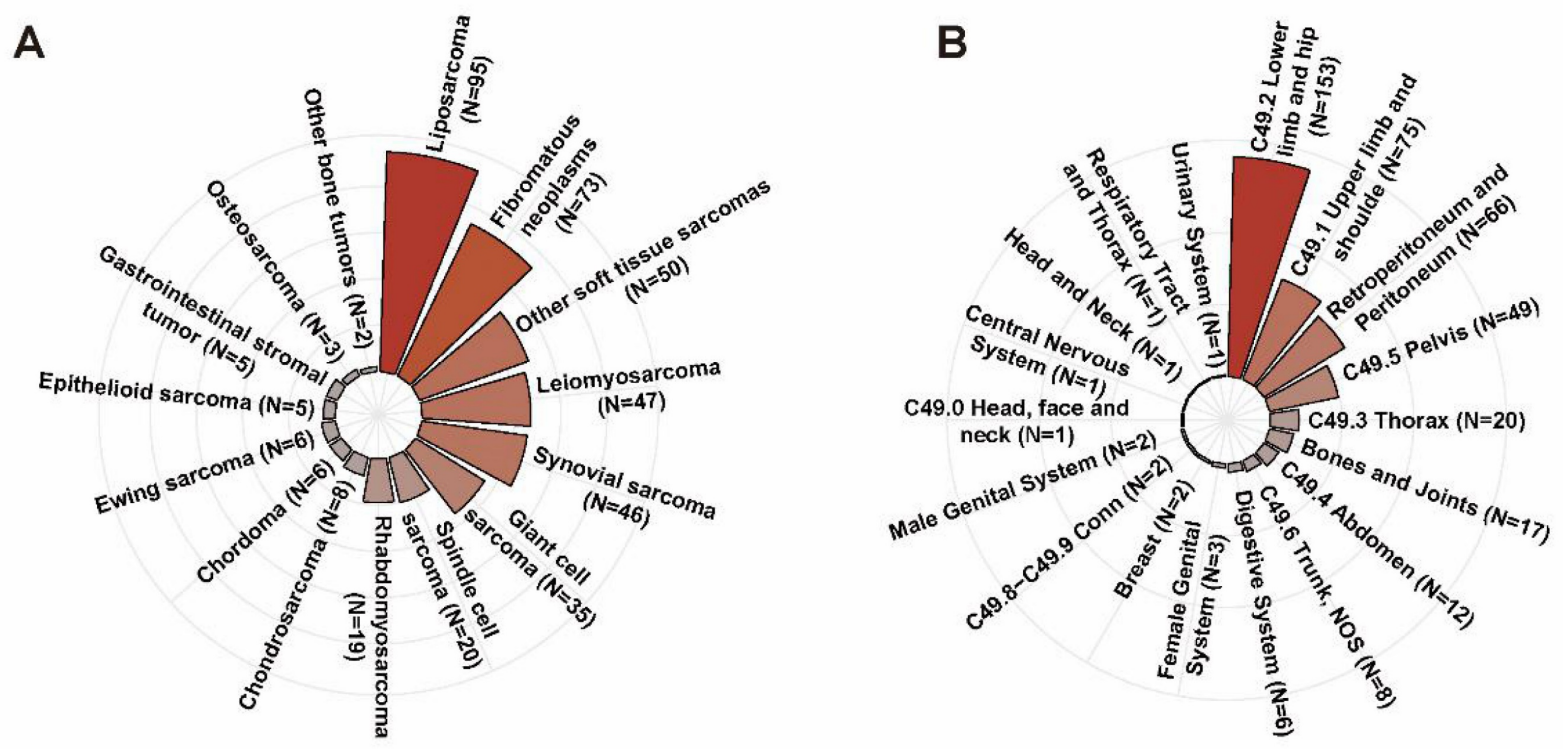
The predominant histologic subtypes (A) and the tumor sites (B) in IORT group.

In the IORT group, the mean age of patients was 51.7±20.7 years. There were 235 male patients (56.0%) and 185 female cases (44.0%), respectively. The majority of patients were White race (N=341, 81.2%) and nearly half of them were diagnosed with localized disease (N=205, 48.8%). As shown in Figure 3A, the predominant histologic subtypes in the IORT group were liposarcoma (N=95, 22.6%), fibromatous neoplasms (N=73, 17.4%), leiomyosarcoma (N=47, 11.2%), synovial sarcoma (N=46, 11.0%) and giant cell sarcoma (N=35, 8.3%). Lower limb and hip (N=153, 36.4%), upper limb and shoulder (N=75, 17.9%), retroperitoneum and peritoneum (N=66, 15.7%) were the common tumor sites in the IORT group as described in Figure 3B. The number of patients who received neoadjuvant/adjuvant chemotherapy was 149, accounting for 35.5% in the IORT group. As for radiotherapy modes in the IORT group, 190 of 420 cases received IORT alone (IORT- group) while the other 230 patients received the combination of IORT and neoadjuvant/ adjuvant RT (IORT+ group). As presented in Supplementary Table 1, the proportion of patients receiving chemotherapy was significantly higher in the IORT+ group compared to the IORT- group (P = 0.003). No significant differences were observed between the two groups in terms of other variables.

### Survival outcome and subgroup analyses

**Figure 4:**
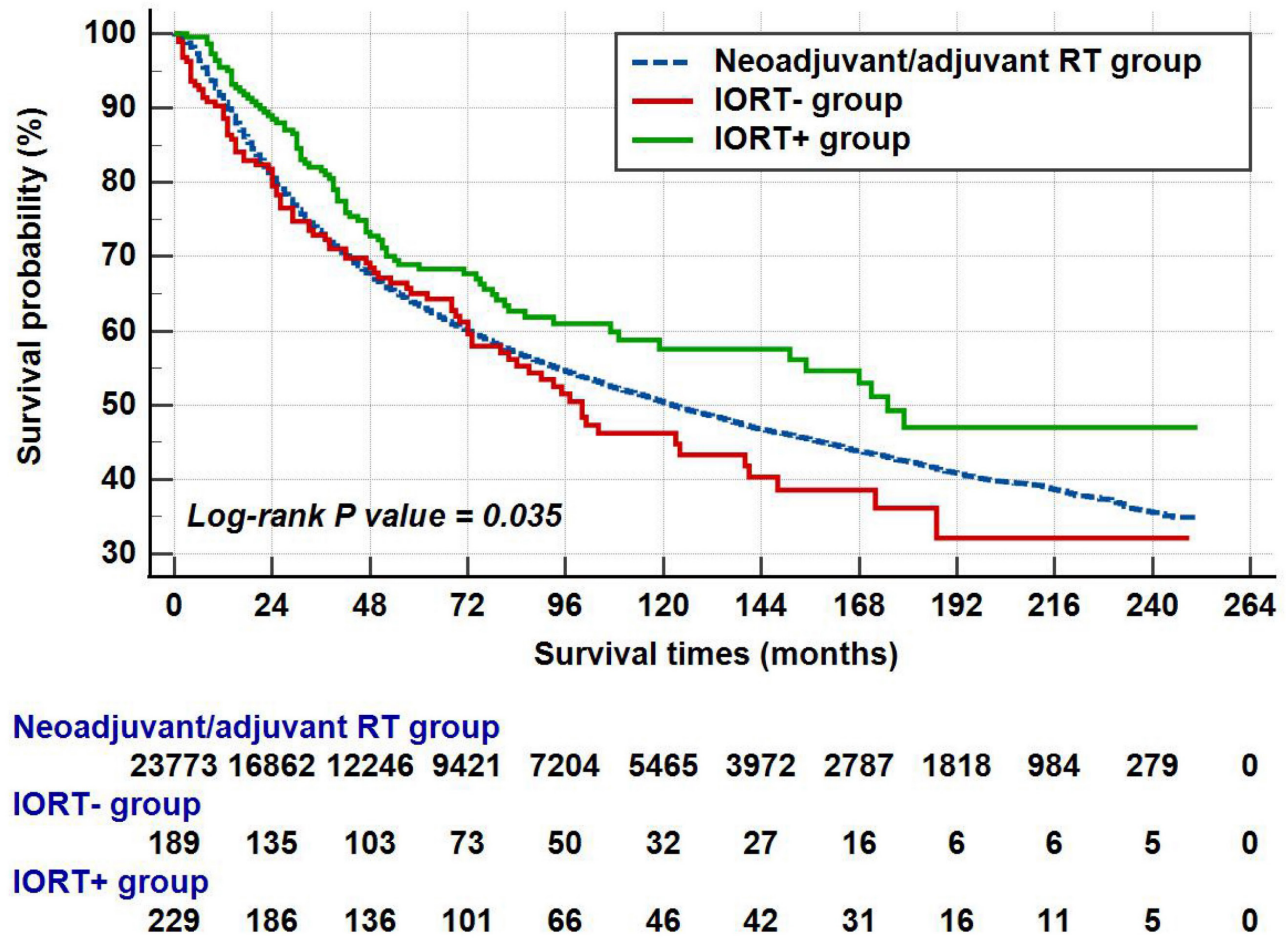
The survival times of different groups. Note: The patient counts in this figure were lower than the total numbers reported in the Abstract and other sections because patients with a survival time of less than one month were not shown. Specifically, 104 patients from the neoadjuvant/adjuvant RT group and one patient each from the IORT- and IORT+ groups were excluded.

The median survival of all patients was 122.0 (95%CI: 117.1-126.9) months. In the neoadjuvant/adjuvant RT group, the mean survival and median survival time were 136.2 (95%CI: 134.6-137.7) and 122.0 (95%CI: 117.1-126.9) months, respectively. As for IORT group, the median survival time was up to 141.0 (95%CI: 101.1-180.9) months and the 1-year, 3-year, 5-year and 10-year OS rates were 92.4%, 77.7%, 66.9% and 52.3%, respectively. As shown in Figure 4, patients in the IORT+ group presented better survival outcome when compared with the neoadjuvant/adjuvant RT group and the IORT- group (P=0.035). The survival outcome of these three groups was summarized in Table 2.

**Figure 5:**
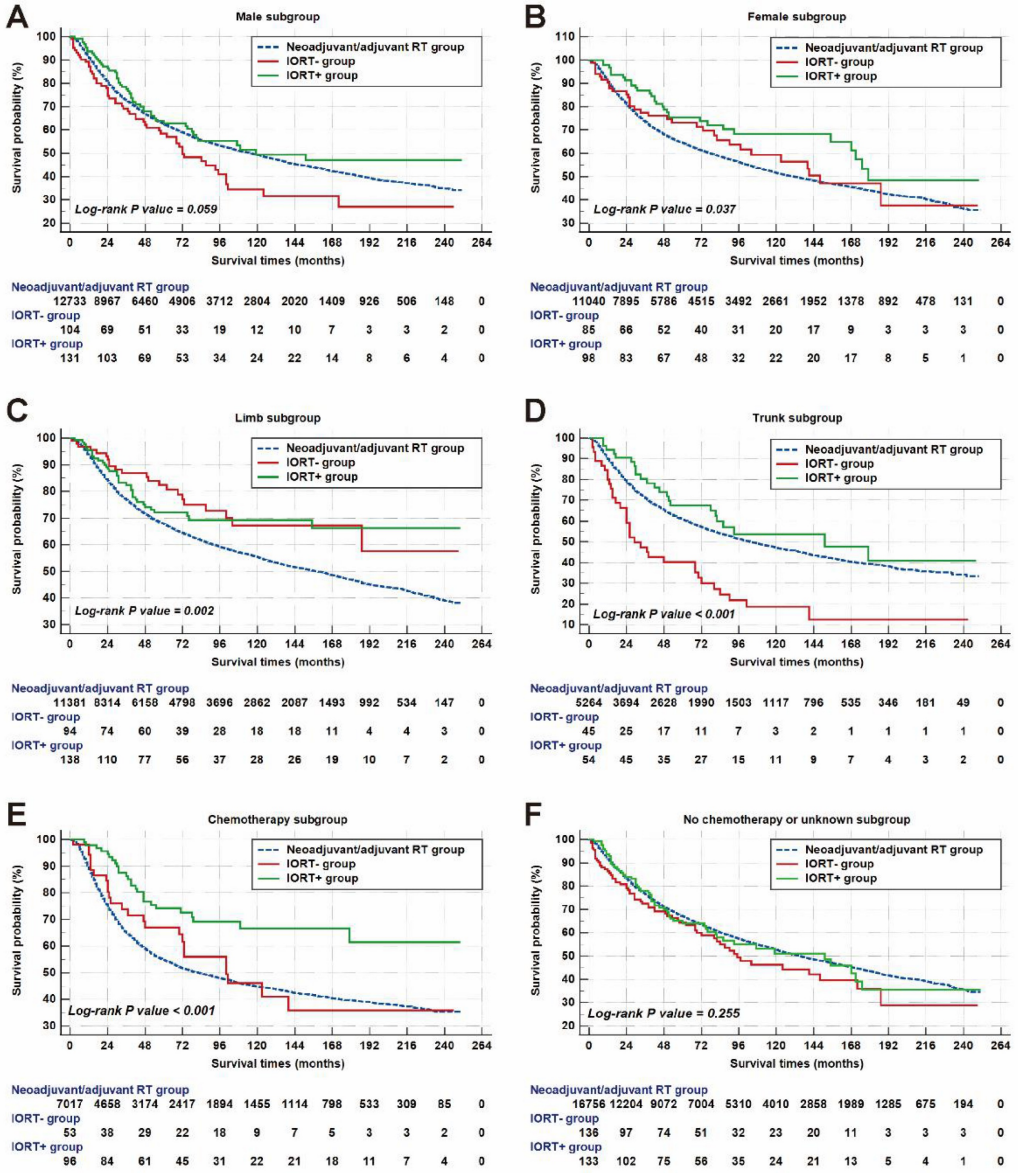
Subgroup analyses of survival times in different groups, stratified by gender, primary tumor location, and chemotherapy. Note: The patient counts in this figure were lower than the total numbers reported in the Abstract and other sections because patients with a survival time of less than one month were not shown.

In male patients, the median survival in the neoadjuvant/adjuvant RT group, IORT+ group, and the IORT- group were 116.0 (95%CI: 109.9-122.1), 72.0 (95%CI: 50.0-94.0) and 119.0 (95%CI: not available) months, respectively. As presented in Figure 5A, the difference between these groups was not statistically significant. In female, the median survival of the IORT+ group (179.0 months, 95%CI: not available) was higher than those in the neoadjuvant/adjuvant RT group (131.0 months, 95%CI: 123.1-138.9 months) and IORT- group (148.0 months, 95%CI: 98.4-197.6 months). The survival curve of female patients was shown in Figure 5B (P = 0.037).

As for patients with tumors located on limb, the median survival of the neoadjuvant/ adjuvant RT group was 158.0 (95%CI: 149.6-166.4) months, which was shorter than those in the IORT- group and IORT+ group (Figure 5C, P = 0.002). In trunk subgroup, there was a statistically significant difference in survival outcome between groups with a P value < 0.001 as shown in Figure 5D. The median survival in the neoadjuvant/adjuvant RT group, the IORT- group and the IORT+ group were 103.0 (95%CI: 94.4-111.6) months, 29.0 (95%CI: 16.4-41.6) months and 151.0 (95%CI: 50.7-251.3) months, respectively.

For musculoskeletal malignancy patients who received the performance of chemotherapy, the mean survival of cases in the IORT+ group was up to 178.3 (95%CI: 155.9-200.6) months and no more than half of patients died until the last follow-up. Meanwhile, the mean survival of the neoadjuvant/adjuvant RT group and the IORT- group were 125.2 (95%CI: 122.4-128.9) and 127.4 (95%CI: 96.7-158.0) months, respectively. Patients in the IORT+ group had a longer survival time than the other two groups, as shown in Figure 5E. However, there were no significant differences between radiotherapy treatment groups in patients who did not receive chemotherapy or those without specific chemotherapy information (Figure 5F).

To further investigate the potential survival benefit, we conducted a subgroup analysis based on histological types. As illustrated in Supplementary Figure 1, the analysis compared the survival outcomes across three treatment modalities (neoadjuvant/adjuvant RT group, IORT- group, and IORT+ group) within both soft tissue sarcoma and bone sarcoma subgroups. The results showed no statistically significant differences in survival outcomes among the three groups in either the soft tissue sarcoma or bone sarcoma subgroups. However, in the soft tissue sarcoma subgroup (Supplementary Figure 1A), the IORT+ group demonstrated a longer mean survival time (153.2 months) compared to the neoadjuvant/adjuvant RT group (135.1 months) and the IORT- group (125.4 months). Meanwhile, the mean survival of the neoadjuvant/adjuvant RT group, the IORT- group and the IORT+ group in bone sarcoma subgroup were 143.4 (95%CI: 139.1-147.7), 117.1 (95%CI: 58.9-175.3) months and 127.9 (95%CI: 100.8-154.9) months, respectively (P =0.301).

### Prognostic factors of patients with IORT

The Cox proportional hazards regression analysis was performed to identify the independent prognostic factors of musculoskeletal malignancy patients who underwent IORT. In the univariate analysis, age at diagnosis, gender, primary tumor site, tumor Grade, SEER stage, T stage, N stage, M stage, bone metastasis, radiotherapy method (IORT alone or IORT plus neoadjuvant/adjuvant RT), and the performance of chemotherapy were associated with patients' survival outcome. To adjust these parameters and exclude potential confounders, the multivariate analysis was performed and then several independent prognostic factors were identified. Older age (versus younger age; HR=1.02, 95% CI: 1.01-1.03, P<0.001), tumor located in the trunk (versus limb tumor location; HR=1.95, 95% CI: 1.33-2.85, P=0.001) and other sites (versus limb tumor location; HR=1.70, 95% CI: 1.10-2.61, P=0.016), higher tumor Grade (versus Grade I; Grade III: HR=3.06, 95% CI: 1.58-5.90, P=0.001; Grade IV: HR=3.63, 95% CI: 1.97-6.70, P<0.001), regional SEER stage (versus localized SEER stage; HR=2.11, 95% CI: 1.38-3.23, P=0.001) were independent prognostic factors for worse survival. Meanwhile, female gender (versus male gender; HR=0.56, 95% CI: 0.40-0.79, P=0.001) was an independent prognostic factor for better survival. Besides, higher T stage and N stage were proved to indicate worse survival when compared with their counterparts. The performance of chemotherapy was a protective factor with a 0.66-fold increased risk of death (versus No/Unknown chemotherapy; 95% CI: 0.44-1.00, P=0.048). It is worth noting that the combination of IORT and neoadjuvant/adjuvant RT presented a survival benefit for patients. There was a 0.65-fold of risk death when compared with IORT alone. Detailed information about Cox regression analysis was listed in Table 3.

## Discussion

The treatment in musculoskeletal malignancy usually adopts a comprehensive model emphasizing interdisciplinary collaboration, in which surgery is the cornerstone of therapy while auxiliary radiation therapy servers as an effective mean to improve local tumor control 19. As for IORT, two major approaches have been reported and used worldwide, thus IOERT and high-dose-rate brachytherapy. Johannes *et al.* summarized the previous literature about the application of brachytherapy for STS and analyzed the recurrence rate as well as local complications 20. It reported a local control rate of 50%-90% in STS of the extremities after brachytherapy as monotherapy 20. In another study, the perioperative high-dose-rate interstitial brachytherapy (PHDRIBT) was performed in localized STS patients after two days of tumor resection and EBRT was supplemented after one month 21. No recurrent cases were observed during a median follow-up of 46.0 months and the 5-year disease-free survival rate was reported to be 63.0% 21. Both excellent local control and high survival rate could be acquired in this treatment regime involving both PHDRIBT and EBRT 21. To validate the advancements of IORT treatment, we conducted a retrospective study based on the SEER database to explore the effect of IORT in musculoskeletal malignancy. Unlike previous studies that have focused on specific clinical situations or isolated surgical challenges, our research systematically explored the application of IORT across different contexts within the musculoskeletal malignancy. We found that patients with the combination treatment of IORT and neoadjuvant/adjuvant RT presented a median survival time up to 175.0 months, which was better than that of patients who underwent IORT alone or neoadjuvant/adjuvant RT alone. Besides, the potential prognostic factors of patients with IORT treatment were identified in our study.

IORT is performed under the condition of fully exposing the irradiated area during surgery. It helps to set the irradiation area accurately and eliminates the time interval between surgical resection and postoperative radiation therapy 22. During the surgical procedure, the normal tissues and radiosensitive organs surrounding to the target area could be removed or temporarily shielded, ensuring the adequate target dose to achieve local control 22. Furthermore, it has been reported that a single high-dose irradiation during surgery could yield a 2.5-fold greater biological effect than that of conventional external irradiation 23. Azinovic *et al.* conducted the one of the earliest IORT study in 2003 and 45 patients with extremity sarcomas were analyzed, including 19 patients with recurrent disease 24. Most of them (36/45, 80.0%) were diagnosed with tumor larger than 5.0cm and nine patients relapsed until the last follow-up with an actuarial local control rate at 5-year of 88.0%. In Austria, a total of 35 patients with high-grade STS were retrospectively analyzed 25. All of them received IORT treatment during limb-preserving surgery, and pre/postoperative radiotherapy was also performed 25. At the last follow-up, the 2-year local control rate was up to 94.3% while the local recurrence rate for R0, R1 and R2 resections were 6.0%, 13.0% and 100.0%, respectively 25. It concluded that the combination of IORT and pre- or postoperative radiotherapy could help for satisfactory local tumor control 25.

The American Society for Radiation Oncology (ASTRO) and the European Society of Radiotherapy & Oncology (ESTRO) have elaborated the role and advantages of IORT and proposed expert recommendation on the application of IORT 26, 27. The ASTRO recommend that IORT should be used in conjunction with auxiliary external irradiation to limit local recurrence 26. IORT is suitable for the situation in which the surgical margin may be positive. The dose range of IORT should be 10-17.5 Gy in the abdomen and 10-20 Gy in the limbs 26. It is also recommended that the higher dose among appropriate doses should be used in selected cases with high risk of positive margin 26. On this basis, ESTRO has claimed that the combination of preoperative RT and IORT could be more advantageous than postoperative RT alone in controlling local tumors and avoiding late-stage toxicities 27. In addition, to prevent severe neurotoxic reaction, the dose of IORT should be limited to below 12.5Gy 27.

Several demographic and clinicopathological characteristics, including age, tumor Grade, clinical stage, resection margin, were found to be associated with the survival outcome of patients receiving IORT treatment in the previous studies 18, 28, 29. In a single-center retrospective study conducted by the University of Heidelberg, a total of 183 patients with extremity STS were treated with IOERT and preceded/followed EBRT. After a median follow-up of 64.0 months, the 5-year OS and 10-year OS rate were 77.0% and 66.0%, respectively 28. In univariate analysis, tumor Grade, metastases prior/at the time of IOERT, and clinical stage were three factors associated with OS. Further multivariate analysis concluded that tumor Grade and distant metastasis were two prognostic factors affecting survival outcome 28. Our current findings based on SEER cohort were consistent with, and extended, the previously study. We found tumor location was associated with patients' OS while female gender and the performance of chemotherapy indicated good survival outcome. Patients with tumor located in trunk had a 1.95-fold risk of mortality compared with patients with limb-located tumors. The potential cause behind the effect of tumor location on survival might be attributed to tumor resectability in different sites.

Our study was limited by the biases arising from the SEER database and the retrospective design of study. Firstly, the end point of our study was restricted to OS since neither disease-free survival (DFS) after IORT nor local control rate was recorded in the SEER database. This limitation cannot be ignored considering the fact that IORT is an efficient method for local tumor control. Second, the information on IORT dose, resection margins as well as adjacent anatomical relationships was not available in the SEER database. These variables were significantly associated with the survival outcome of patients receiving IORT treatment 18. However, our findings may not provide effective guidance for addressing specific clinical challenges, such as tendon involvement 30 and soft tissue reconstruction 31. Finally, the histologic subgroup analysis was not performed adequately. Based on the classification criteria existing in the SEER database, we found no statistically significant result in our Cox proportional hazards regression analysis. Thus, further large-scale clinical studies are needed to validate the results.

## Conclusions

The key strength of our study was the comprehensive analysis conducted on a large cohort derived from the SEER database, encompassing a broad range of musculoskeletal malignancy. This approach allowed us to evaluate the impact of intraoperative radiotherapy (IORT) across various tumor types and clinical scenarios, offering a more generalized understanding of its effectiveness in diverse patient populations. Despite the aforementioned limitations, we confirmed the survival benefit of the combination of IORT during surgery and neoadjuvant/adjuvant RT. Furthermore, we identified several independent prognostic factors of patients undergoing IORT treatment, which can aid in clinically making individualized treatment plans.

## Supplementary Material

Supplementary figure and table.

## Figures and Tables

**Figure 1:**
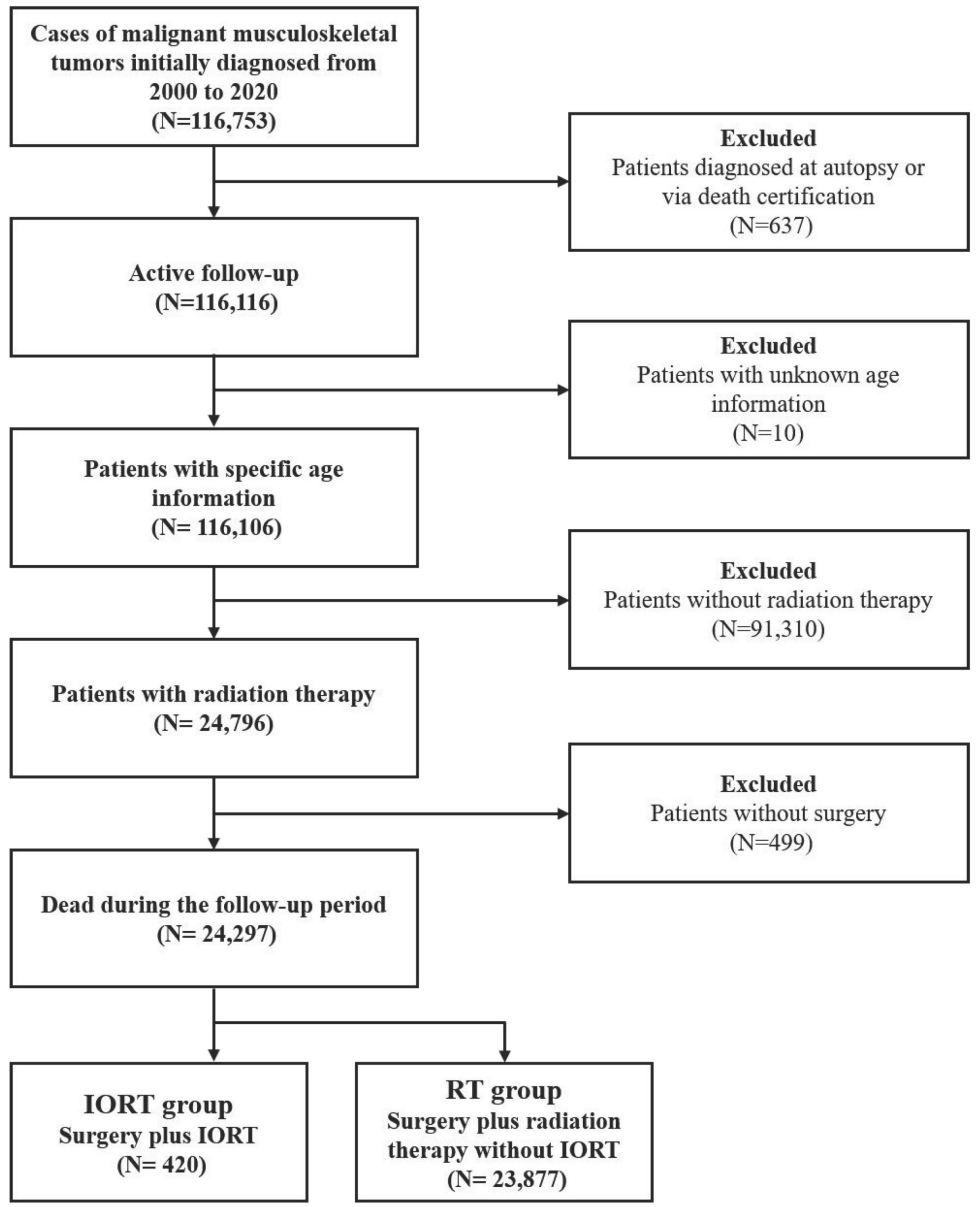
The flowchart of cohort selection.

**Table 1:** Demographic and clinicopathological characteristics of patients with musculoskeletal malignancy in SEER database.

Characteristics	Neoadjuvant/adjuvant RT group	IORT group	P-value
**Gender**			
Male	12795 (53.6)	235 (56.0)	*0.335*
Female	11082 (46.4)	185 (44.0)	
**Marital status**			
Unmarried	9801 (41)	181 (43.1)	*0.151*
Married	13289 (55.7)	232 (55.2)	
Unknown	787 (3.3)	7 (1.7)	
**Race**			
White	19243 (80.6)	341 (81.2)	*<0.001*
Black	2337 (9.8)	20 (4.8)	
Others	2193 (9.2)	59 (14.0)	
Unknown	104 (0.4)	0 (0)	
**Median household income**			
< $65,000	6874 (28.8)	100 (23.8)	*<0.001*
$65,000 - $75,000	6331 (26.5)	53 (12.6)	
> $75,000	10670 (44.7)	267 (63.6)	
Unknown	2 (0)	0 (0)	
**Histology**			
Osteosarcoma	417 (1.7)	3 (0.7)	*<0.001*
Chondrosarcoma	777 (3.3)	8 (1.9)	
Ewing sarcoma	878 (3.7)	6 (1.4)	
Fibromatous neoplasms	4473 (18.7)	73 (17.4)	
Liposarcoma	4258 (17.8)	95 (22.6)	
Synovial sarcoma	1391 (5.8)	46 (11.0)	
Leiomyosarcoma	3222 (13.5)	47 (11.2)	
Rhabdomyosarcoma	1172 (4.9)	19 (4.5)	
Gastrointestinal stromal tumor	69 (0.3)	5 (1.2)	
Spindle cell sarcoma	879 (3.7)	20 (4.8)	
Epithelioid sarcoma	283 (1.2)	5 (1.2)	
Desmoplastic small round cell tumor	80 (0.3)	0 (0)	
Chordoma	703 (2.9)	6 (1.4)	
Giant cell sarcoma	2172 (9.1)	35 (8.3)	
Other soft tissue sarcomas	2913 (12.2)	50 (11.9)	
Other bone tumors	190 (0.8)	2 (0.5)	
**Primary site**			
Limb	11432 (47.9)	232 (55.2)	*<0.001*
Trunk	5286 (22.1)	101 (24.0)	
Head, face, neck	1796 (7.5)	2 (0.5)	
Other sites	5142 (21.5)	83 (19.8)	
Unknown	221 (0.9)	2 (0.5)	
**Grade**			
Well differentiated; Grade I	1790 (7.5)	51 (12.1)	*<0.001*
Moderately differentiated; Grade II	3345 (14.0)	84 (20.0)	
Poorly differentiated; Grade III	5208 (21.8)	109 (26.0)	
Undifferentiated; anaplastic; Grade IV	6941 (29.1)	105 (25.0)	
Unknown	6593 (27.6)	71 (16.9)	
**SEER Stage**			
Localized	12321 (51.6)	205 (48.8)	*0.121*
Regional	5434 (22.8)	116 (27.6)	
Distant	1935 (8.1)	29 (6.9)	
Unknown	4187 (17.5)	70 (16.7)	
**T stage**			
T1	5256 (22.0)	86 (20.5)	*<0.001*
T2	9775 (40.9)	226 (53.8)	
T3	737 (3.1)	9 (2.1)	
T4	526 (2.2)	5 (1.2)	
Unknown	7583 (31.8)	94 (22.4)	
**N stage**			
N0	16291 (68.2)	315 (75.0)	*<0.001*
N1	662 (2.8)	19 (4.5)	
Unknown	6924 (29.0)	86 (20.5)	
**M stage**			
M0	16145 (67.6)	312 (74.3)	*0.003*
M1	1296 (5.4)	26 (6.2)	
Unknown	6436 (27.0)	82 (19.5)	
**Bone metastasis**			
No	13190 (55.2)	245 (58.3)	*0.423*
Yes	293 (1.2)	4 (1.0)	
Unknown	10394 (43.5)	171 (40.7)	
**Brain metastasis**			
No	13426 (56.2)	250 (59.5)	*0.269*
Yes	53 (0.2)	0 (0)	
Unknown	10398 (43.5)	170 (40.5)	
**Liver metastasis**			
No	13361 (56.0)	246 (58.6)	*0.170*
Yes	110 (0.5)	4 (1)	
Unknown	10406 (43.6)	170 (40.5)	
**Lung metastasis**			
No	12878 (53.9)	242 (57.6)	*0.284*
Yes	594 (2.5)	8 (1.9)	
Unknown	10405 (43.6)	170 (40.5)	
**Chemotherapy**			
No/Unknown	16837 (70.5)	271 (64.5)	*0.008*
Yes	7040 (29.5)	149 (35.5)	

**Table 2:** The survival outcome of musculoskeletal malignancy patients who received radiation therapy in SEER database.

Groups	Mean survival	95%CI	Median survival	95% CI	1-year OS%	3-year OS%	5-year OS%	10-year OS%
Neoadjuvant/adjuvant RT	136.2	134.6 - 137.7	122.0	118.0 - 127.0	90.8%	73.0%	63.4%	50.3%
IORT-	125.8	108.8 - 142.8	100.0	73.0 - 141.0	88.6%	72.9%	65.0%	46.2%
IORT+	155.2	139.7 - 170.8	175.0	119.0 - 179.0	95.5%	81.6%	68.3%	57.6%

**Table 3:** Identifying the independent prognostic factors of patients underwent IORT in SEER database.

Characteristics	Univariate		Multivariate	
	HR (95% CI)	*P-Value*	HR (95% CI)	*P-Value*
**Age at diagnosis**	1.03 (1.02-1.04)	*<0.001*	1.02 (1.01-1.03)	*<0.001*
**Gender**				
Male	1.00 (Reference)		1.00 (Reference)	
Female	0.65 (0.48-0.89)	*0.006*	0.56 (0.40-0.79)	*0.001*
**Marital status**				
Unmarried	1.00 (Reference)			
Married	1.03 (0.76-1.40)	*0.856*		
Unknown	1.55 (0.56-4.24)	*0.397*		
**Race**				
White	1.00 (Reference)			
Black	0.58 (0.24-1.42)	*0.236*		
Others	1.36 (0.91-2.05)	*0.135*		
**Median household income**				
< $65,000	1.00 (Reference)			
$65,000 - $75,000	0.97 (0.59-1.59)	*0.900*		
> $75,000	0.82 (0.58-1.16)	*0.264*		
**Histology**				
Osteosarcoma	1.00 (Reference)			
Chondrosarcoma	1.16 (0.12-11.13)	0.900		
Ewing sarcoma	5.85 (0.68-50.25)	0.107		
Fibromatous neoplasms	2.15 (0.29-15.75)	0.450		
Liposarcoma	1.87 (0.26-13.64)	0.535		
Synovial sarcoma	0.56 (0.07-4.47)	0.583		
Leiomyosarcoma	2.89 (0.39-21.32)	0.297		
Rhabdomyosarcoma	1.21 (0.15-10.08)	0.859		
Gastrointestinal stromal tumor	1.59 (0.17-15.33)	0.687		
Spindle cell sarcoma	1.52 (0.19-12.37)	0.696		
Epithelioid sarcoma	1.62 (0.15-17.86)	0.695		
Chordoma	0.62 (0.04-9.89)	0.733		
Giant cell sarcoma	1.31 (0.16-10.53)	0.797		
Other soft tissue sarcomas	2.19 (0.30-16.28)	0.442		
Other bone tumors	-	0.952		
**Primary site**				
Limb	1.00 (Reference)		1.00 (Reference)	
Trunk	2.60 (1.81-3.75)	*<0.001*	1.95 (1.33-2.85)	*0.001*
Head, face, neck	1.46 (0.20-10.53)	0.709	0.75 (0.09-6.22)	*0.792*
Other sites	2.52 (1.73-3.67)	*<0.001*	1.70 (1.10-2.61)	*0.016*
Unknown	4.82 (1.17-19.81)	0.029	3.96 (0.90-17.47)	*0.070*
**Grade**				
Well differentiated; Grade I	1.00 (Reference)		1.00 (Reference)	
Moderately differentiated; Grade II	0.85 (0.43-1.69)	*0.642*	1.44 (0.69-3.02)	*0.331*
Poorly differentiated; Grade III	1.86 (1.04-3.34)	*0.036*	3.06 (1.58-5.90)	*0.001*
Undifferentiated; anaplastic; Grade IV	2.60 (1.47-4.58)	*0.001*	3.63 (1.97-6.70)	*<0.001*
Unknown	1.51 (0.81-2.80)	*0.196*	2.62 (1.32-5.17)	*0.006*
**SEER Stage**				
Localized	1.00 (Reference)		1.00 (Reference)	
Regional	2.74 (1.86-4.04)	*<0.001*	2.11 (1.38-3.23)	*0.001*
Distant	3.45 (2.02-5.88)	*<0.001*	0.84 (0.11-6.58)	*0.865*
Unknown	2.34 (1.55-3.52)	*<0.001*	0.93 (0.43-2.04)	*0.861*
**T stage**				
T1	1.00 (Reference)		1.00 (Reference)	
T2	3.22 (1.80-5.75)	*<0.001*	2.06 (1.11-3.83)	*0.022*
T3	3.54 (0.46-27.32)	*0.226*	2.74 (0.33-22.78)	*0.352*
T4	1.63 (0.21-12.50)	*0.636*	0.28 (0.03-2.45)	*0.252*
Unknown	3.85 (2.11-7.03)	*<0.001*	2.17 (0.73-6.46)	*0.165*
**N stage**				
N0	1.00 (Reference)		1.00 (Reference)	
N1	2.34 (1.25-4.36)	*0.008*	2.06 (1.11-3.83)	*0.022*
Unknown	1.76 (1.26-2.44)	*0.001*	2.74 (0.33-22.78)	*0.352*
**M stage**				
M0	1.00 (Reference)		1.00 (Reference)	
M1	2.73 (1.65-4.57)	*<0.001*	4.35 (0.52-36.34)	*0.175*
Unknown	1.75 (1.25-2.45)	*0.001*	1.03 (0.22-4.82)	*0.971*
**Bone metastasis**				
No	1.00 (Reference)		1.00 (Reference)	
Yes	5.75 (2.08-15.86)	*0.001*	1.88 (0.56-6.38)	*0.310*
Unknown	1.57 (1.14-2.18)	*0.007*	1.25 (0.84-1.86)	*0.270*
**Brain metastasis**				
No	1.00 (Reference)			
Unknown	1.47 (1.07-2.03)	*0.018*		
**Liver metastasis**				
No	1.00 (Reference)			
Yes	2.51 (0.79-8.00)	*0.119*		
Unknown	1.52 (1.10-2.10)	*0.012*		
**Lung metastasis**				
No	1.00 (Reference)			
Yes	1.81 (0.66-4.97)	*0.250*		
Unknown	1.51 (1.09-2.10)	*0.013*		
**Radiotherapy method**				
IORT only	1.00 (Reference)		1.00 (Reference)	
IORT plus neoadjuvant/adjuvant RT	0.70 (0.52-0.94)	*0.017*	0.65 (0.47-0.90)	*0.009*
**Chemotherapy**				
No/Unknown	1.00 (Reference)		1.00 (Reference)	
Yes	0.69 (0.50-0.95)	*0.023*	0.66 (0.44-1.00)	*0.048*
