# Supplementary material for: The effect of intraoperative radiotherapy in musculoskeletal malignancy: A population study from US SEER database
Source: J Cancer. 2025 Jan 1;16(1):135–45. doi: 10.7150/jca.100678 (PMC11660128; doi:10.7150/jca.100678)
Supplement: Supplementary file 1 — Supplementary figure and table. [file jcav16p0135s1.pdf]

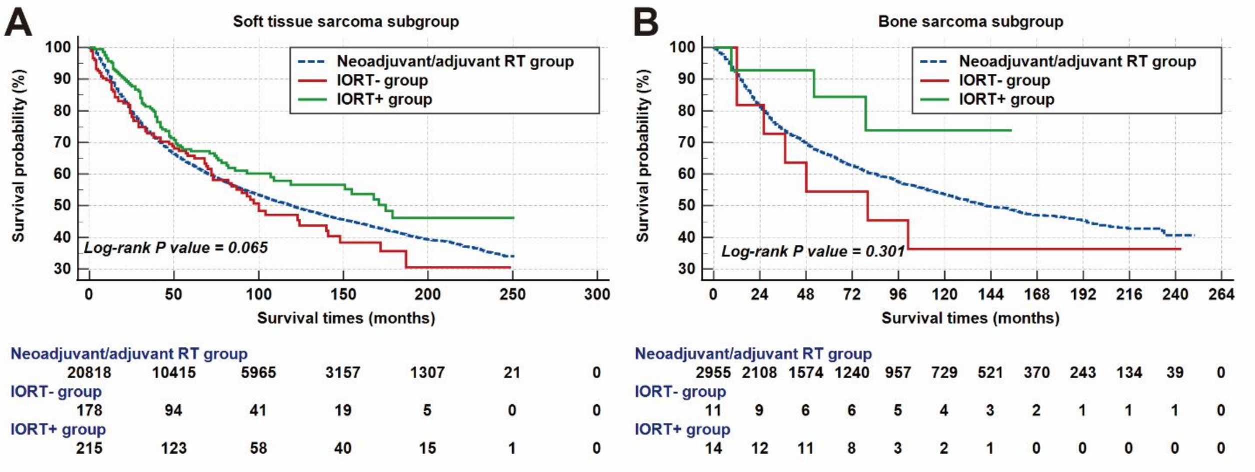

Supplementary Figure 1 Subgroup analyses of survival times in different groups, stratified by histological types.

Supplementary Table 1. Demographic and clinicopathological characteristics of patients with the treatment of IORT alone (IORT- group) or the combination of IORT and neoadjuvant/ adjuvant RT (IORT+ group).

Characteristics	IORT- group	IORT+ group	*P-value*
**Gender**			
Male	104 (54.7)	131 (57)	*0.648*
Female	86 (45.3)	99 (43)	
**Marital status**			
Unmarried	85 (44.7)	96 (41.7)	
Married	100 (52.6)	132 (57.4)	*2.582*
Unknown	5 (2.6)	2 (0.9)	
**Race**			
White	152 (80)	189 (82.2)	
Black	11 (5.8)	9 (3.9)	*0.836*
Others	27 (14.2)	32 (13.9)	
Unknown	0 (0)	0 (0)	
**Median household income**			
< $65,000	37 (19.5)	63 (27.4)	
$65,000 - $75,000	33 (17.4)	20 (8.7)	*8.951*
> $75,000	120 (63.2)	147 (63.9)	
Unknown	0 (0)	0 (0)	
**Histology**			
Osteosarcoma	2 (1.1)	1 (0.4)	
Chondrosarcoma	4 (2.1)	4 (1.7)	
Ewing sarcoma	3 (1.6)	3 (1.3)	
Fibromatous neoplasms	32 (16.8)	41 (17.8)	
Liposarcoma	40 (21.1)	55 (23.9)	
Synovial sarcoma	20 (10.5)	26 (11.3)	
Leiomyosarcoma	28 (14.7)	19 (8.3)	
Rhabdomyosarcoma	7 (3.7)	12 (5.2)	*0.700*
Gastrointestinal stromal tumor	4 (2.1)	1 (0.4)	
Spindle cell sarcoma	8 (4.2)	12 (5.2)	
Epithelioid sarcoma	3 (1.6)	2 (0.9)	
Chordoma	1 (0.5)	5 (2.2)	
Giant cell sarcoma	15 (7.9)	20 (8.7)	
Other soft tissue sarcomas	22 (11.6)	28 (12.2)	
Other bone tumors	1 (0.5)	1 (0.4)	
**Primary site**			
Limb	94 (49.5)	138 (60)	
Trunk	46 (24.2)	55 (23.9)	
Head, face, neck	1 (0.5)	1 (0.4)	*0.064*
Other sites	47 (24.7)	36 (15.7)	
Unknown	2 (1.1)	0 (0)	
**Grade**			

Well differentiated; Grade I	32 (16.8)	19 (8.3)	
Moderately differentiated; Grade II	39 (20.5)	45 (19.6)	
Poorly differentiated; Grade III	45 (23.7)	64 (27.8)	*0.084*
Undifferentiated; anaplastic; Grade IV	42 (22.1)	63 (27.4)	
Unknown	32 (16.8)	39 (17)	
**SEER Stage**			
Localized	90 (47.4)	115 (50)	
Regional	55 (28.9)	61 (26.5)	*0.886*
Distant	12 (6.3)	17 (7.4)	
Unknown	33 (17.4)	37 (16.1)	
**T stage**			
T1	41 (21.6)	45 (19.6)	
T2	94 (49.5)	132 (57.4)	
T3	5 (2.6)	4 (1.7)	*0.533*
T4	2 (1.1)	3 (1.3)	
Unknown	48 (25.3)	46 (20)	
**N stage**			
N0	136 (71.6)	179 (77.8)	
N1	7 (3.7)	12 (5.2)	*0.125*
Unknown	47 (24.7)	39 (17)	
**M stage**			
M0	137 (72.1)	175 (76.1)	
M1	10 (5.3)	16 (7)	*0.298*
Unknown	43 (22.6)	39 (17)	
**Bone metastasis**			
No	108 (56.8)	137 (59.6)	
Yes	1 (0.5)	3 (1.3)	*0.575*
Unknown	81 (42.6)	90 (39.1)	
**Brain metastasis**			
No	110 (57.9)	140 (60.9)	
Yes	0 (0)	0 (0)	*0.536*
Unknown	80 (42.1)	90 (39.1)	
**Liver metastasis**			
No	107 (56.3)	139 (60.4)	
Yes	3 (1.6)	1 (0.4)	*0.375*
Unknown	80 (42.1)	90 (39.1)	
**Lung metastasis**			
No	109 (57.4)	133 (57.8)	
Yes	1 (0.5)	7 (3)	*0.158*
Unknown	80 (42.1)	90 (39.1)	
**Chemotherapy**			
No/Unknown	137 (72.1)	134 (58.3)	*0.003*
Yes	53 (27.9)	96 (41.7)	
